# Chest Pain and Hidden Genetic Risk: A Case Report on the Role of Elevated Lipoprotein(a) in Early Cardiovascular Disease

**DOI:** 10.7759/cureus.100830

**Published:** 2026-01-05

**Authors:** Arowa Abdelgadir, Jimmy Li Voon Chong

**Affiliations:** 1 Internal Medicine, Royal Hampshire County Hospital, Winchester, GBR; 2 Diabetes and Endocrinology, Royal Hampshire County Hospital, Winchester, GBR

**Keywords:** cad: coronary artery disease, familial cardiovascular risk, lifestyle intervention, lipoprotein (a), pcsk9 inhibitors

## Abstract

Lipoprotein(a) (Lp(a)) is increasingly recognized as a significant contributor to the risk of atherosclerotic cardiovascular disease (ASCVD). We report the case of a 53-year-old woman who presented with chest pain and has notable family history of premature cardiovascular events. Investigation revealed a markedly elevated Lp(a) level of 492 nmol/L, alongside the presence of coronary artery disease necessitating stenting.

Despite adherence to high-intensity statin therapy, her low-density lipoprotein (LDL) cholesterol levels remained suboptimal. Consequently, we initiated treatment with a PCSK9 inhibitor to achieve further reductions in LDL cholesterol. This case underscores the importance of routinely measuring Lp(a), as recommended by European guidelines, which advocate for its assessment at least once during adulthood for effective risk stratification.

While lifestyle interventions play a critical role in cardiovascular health, targeted therapies such as PCSK9 inhibitors and emerging nucleic acid-based treatments, including Zerlasiran, offer promising options for significantly lowering Lp(a) levels. Recognizing and addressing elevated Lp(a) is vital for identifying patients at high cardiovascular risk and for informing tailored management strategies aimed at improving patient outcomes.

## Introduction

Lipoprotein(a) (Lp(a)) is a genetically influenced lipoprotein composed of a low-density lipoprotein (LDL)-like particle covalently linked to apolipoprotein(a) (apo(a)). Apo(a) contains multiple kringle domains, including the highly polymorphic kringle IV type 2 (KIV-2) repeat region. The number of KIV-2 repeats strongly affects apo(a) synthesis, as smaller apo(a) isoforms with fewer repeats are produced more efficiently and therefore lead to higher plasma Lp(a) levels [1].

This genetic variability has important clinical implications. Elevated Lp(a) is increasingly recognized as a significant cardiovascular risk factor, and large epidemiological studies have shown considerable differences in its prevalence across populations. For example, the INTERASPIRE study reported that Lp(a) ≥150 nmol/L occurs in 4-26% of patients with established coronary artery disease, highlighting both its relevance and global variability [2].

Given this variability and the strong association between high Lp(a) levels and cardiovascular disease, measuring Lp(a) can be crucial in identifying individuals at increased risk, especially those with a family history of premature cardiovascular disease or unexplained atherosclerosis.

In this report, we describe the case of a 53-year-old woman with chest pain and a strong family history of premature cardiovascular disease who was found to have markedly elevated Lp(a) and severe coronary artery disease requiring stent placement. This case reinforces the importance of incorporating Lp(a) measurement into cardiovascular risk assessment and illustrates how significantly elevated levels can influence clinical decision-making and targeted therapeutic strategies.

## Case presentation

A 53-year-old woman with a body mass index (BMI) of 26 kg/m², who was physically active, a lifelong non-smoker, normotensive, and without diabetes, presented with atypical left-sided chest pain. Her family history was notable for premature coronary artery disease: both siblings had documented elevations in lipoprotein(a), and her father had died from a myocardial infarction in his 50s. The results of the initial lipid assessment at presentation are summarized in Table 1.

**Table 1:** Laboratory data of the patient HDL, high-density lipoprotein

Test	Results	Interpretation of the test results	Reference range
Total cholesterol	6.9 mmol/L	High	0-5.2 mmol/L
HDL cholesterol	2.06 mmol/L	Normal	1.2-10 mmol/L
Total cholesterol /LDL ratio	3.3	Normal	0-4
Troponin level	7.7 ng/L	High	0-5

**Figure 1:**
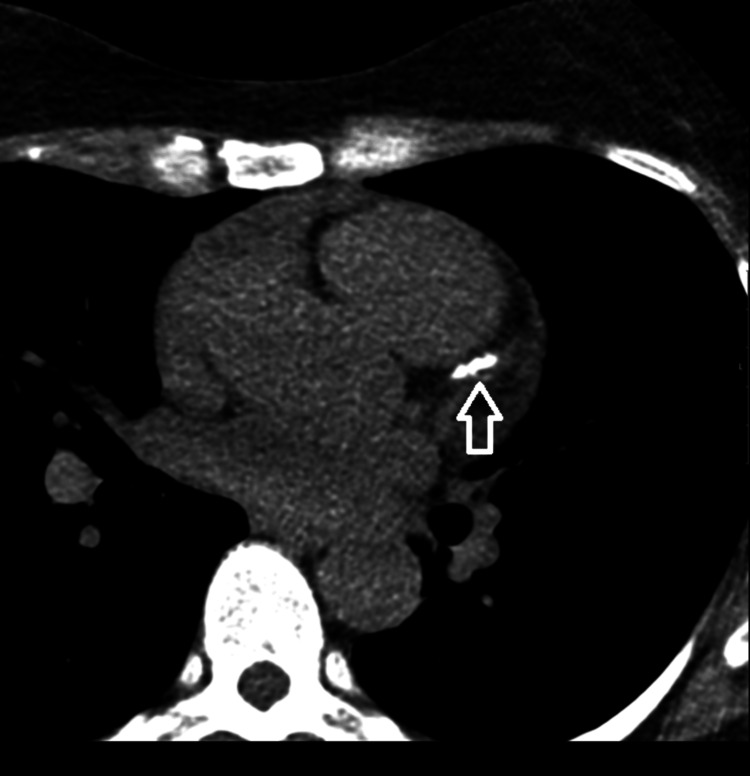
The CT coronary angiogram demonstrates coronary calcification, as indicated by the white arrow.

The patient was reviewed by the cardiology team, who arranged a CT coronary angiogram because of her family history of cardiovascular disease. The CT coronary angiogram scan showed multifocal calcification in all three coronary arteries, with a coronary artery calcium score of 915, which was quite high. There was modest calcification in the dominant right coronary artery (RCA), with mild-to-moderate stenosis due to mixed but predominantly calcific plaque. There was also calcification in the distal left main stem with mild stenosis, and multifocal atheromatous disease in the left anterior descending artery (LAD), with moderate-to-severe stenosis in the mid LAD that appeared partially calcified. Modest calcification was also present in the circumflex artery. Figure 1 illustrates the findings of coronary artery calcification, and Table 2 illustrates the calcium scoring as assessed by CT coronary angiography.

**Table 2:** Calcium scoring as assessed by CT coronary angiography. LM, left main coronary artery; LAD, left anterior descending artery; LCx, left circumflex artery; RCA, right coronary artery

Artery	Lesions	Volume (mm^3^)	Equiv. mass (mg)	Score
LM	1	42.3	9.03	50.5
LAD	3	235.8	56.14	293.5
CX	2	125.9	30.55	153.0
RCA	4	363.2	87.81	418.1
Ca	0	0.0	0.00	0.0
Total	10	767	183.53	915.1
U1	0	0.0	0.00	0.0
U2	0	0.0	0.00	0.0

She was started on aspirin 75 mg once daily, atorvastatin 40 mg once daily at night, and bisoprolol 2.5 mg once daily.

**Figure 2:**
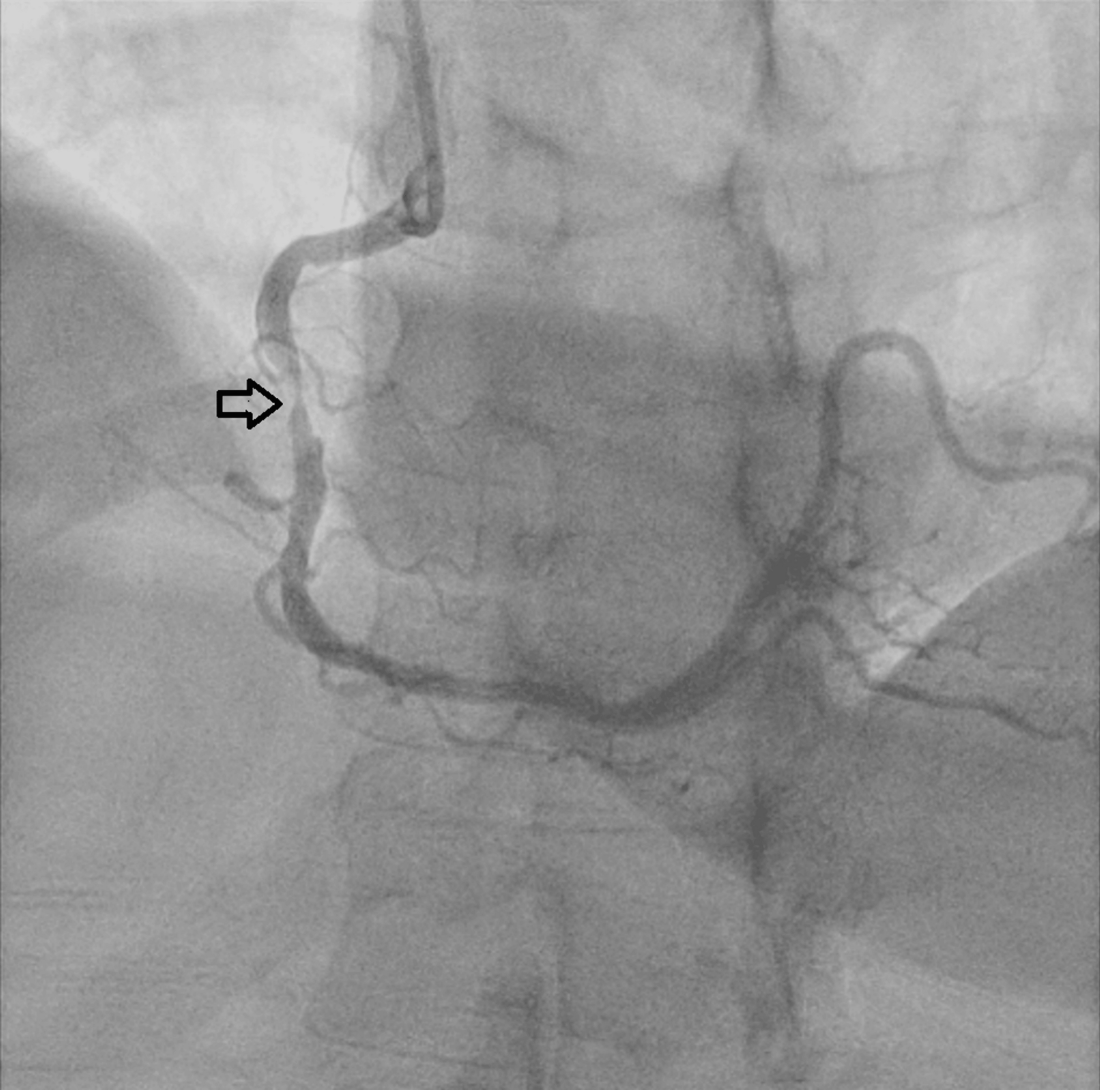
Coronary angiogram demonstrating significant narrowing of the right coronary artery, indicated by the black arrow.

**Figure 3:**
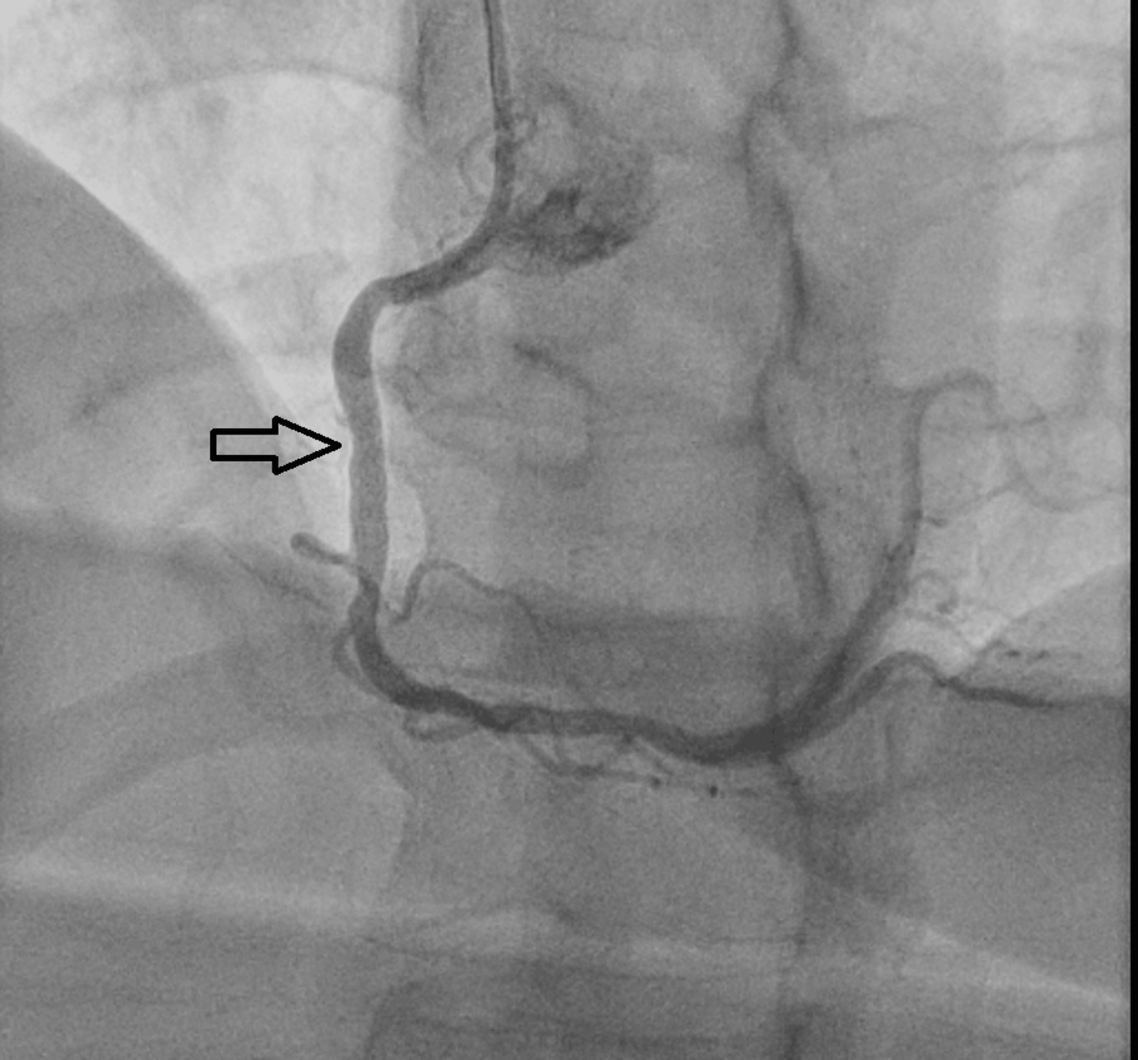
Post-stenting coronary angiogram showing the right coronary artery stent, indicated by the black arrow.

Six weeks later, she was admitted for an elective coronary angiography, during which a stent was successfully placed in the RCA without complications. Figures 2-3 show the coronary angiogram of the RCA before and after stenting, respectively.

She was commenced on dual antiplatelet therapy for one year, after which she remained on aspirin 75 mg once daily. Her atorvastatin dose was also increased to 80 mg once daily.

Given her family history of cardiovascular disease and the fact that both of her siblings had elevated Lp(a) levels, she was screened for Lp(a). Her results are presented in Table 3.

**Table 3:** Laboratory data of the patient. LDL, low-density lipoprotein; HDL, high-density lipoprotein; IHD, ischemic heart disease

Test	Results	Interpretation of the test results	Reference range
Lipoprotein a	492 nmol/L	High	Lipoprotein(a) >32 nmol/L associated with increased risk of IHD
Total cholesterol	4.6 mmol/L	Normal	0-5.2 mmol/L
Triglyceride level	0.68 mmol/L	Normal	0-1.7 mmol/L
HDL cholesterol	2.26 mmol/L	Normal	1.2-10 mmol/L
LDL cholesterol	2.4 mmol/L	High	Target as per guideline <2

Despite being on atorvastatin 80 mg once daily, her LDL cholesterol remained above the optimal target. She was, therefore, started on Praluent (alirocumab) 150 mg, a monoclonal antibody against PCSK9, administered as a subcutaneous injection every two weeks.

At one-year follow-up, her results are presented in Table 4.

**Table 4:** Laboratory data of the patient at one-year follow-up. LDL, low-density lipoprotein; HDL, high-density lipoprotein

Test	Results	Interpretation of the test results	Reference range
Total cholesterol	3.7 mmol/L	Normal	0-5.2 mmol/L
Triglyceride level	0.67 mmol/L	Normal	0-1.7 mmol/L
HDL cholesterol	2.1 mmol/L	Normal	1.2-10 mmol/L
LDL cholesterol	1.3 mmol/L	High	Target as per guideline <2

Following initiation of PCSK9 inhibitor therapy, her LDL cholesterol was reduced by 45.8%. The patient remained asymptomatic, with no subsequent vascular events during follow-up.

## Discussion

Genetic factors account for the vast majority of variation in Lp(a) concentrations. Structurally, Lp(a) consists of an LDL-like particle covalently linked to apolipoprotein(a), which shares considerable homology with plasminogen. This unique composition contributes to the lipoprotein’s pro-atherogenic and pro-thrombotic properties [2].

Supporting its strong heritable nature, data from the UK Biobank demonstrated that elevated Lp(a) was present in 47% of first-degree relatives and 32% of second-degree relatives of individuals with high Lp(a). This pronounced familial clustering highlights the major influence of LPA gene variants and the KIV-2 repeat number on circulating Lp(a) levels, reinforcing the genetic basis of elevated Lp(a) and its relevance to cardiovascular risk [3].

This familial and genetic predisposition carries important clinical consequences. Elevated Lp(a) is well recognized as an independent, causal risk factor for atherosclerotic cardiovascular disease (ASCVD), including coronary artery disease, myocardial infarction, and calcific aortic valve disease. Assessing Lp(a) levels is particularly informative in individuals with premature ASCVD or a strong family history of early cardiac events, as illustrated by our 53-year-old patient. Notably, even when conventional cardiovascular risk factors are optimally managed, genetically high Lp(a) may continue to contribute to residual cardiovascular risk, highlighting the potential role of emerging targeted therapies [4,5].

Worldwide, approximately 20%-25% of individuals have elevated Lp(a), commonly defined as ≥50 mg/dL (≈125 nmol/L), although the prevalence differs substantially between ethnic groups. Among patients with established ASCVD, Lp(a) concentrations ≥150 nmol/L are observed in more than a quarter of cases, emphasizing the benefit of incorporating Lp(a) measurements into risk assessment beyond standard lipid profiles. While genetic determinants are the primary drivers of Lp(a) levels, conditions such as chronic kidney disease, thyroid or liver disorders, pregnancy, menopause, and dietary factors can produce modest additional effects [6].

The clinical importance of elevated Lp(a) is further supported by large epidemiological studies. In the Copenhagen General Population Study, each 50 mg/dL (≈105 nmol/L) increase in genetically determined Lp(a) was associated with a 39% higher risk of peripheral artery disease, demonstrating its strong impact on vascular outcomes. Likewise, the LipidCardio Study reported that patients with suspected or established coronary artery disease and high Lp(a) frequently exhibit more severe and anatomically complex lesions, posing additional challenges for management. Given its strong genetic basis, Lp(a) is largely resistant to conventional lifestyle measures. Dietary modifications, such as substituting saturated fats with carbohydrates or unsaturated fats, can paradoxically increase Lp(a), whereas low-carbohydrate, high-saturated fat diets may produce modest reductions but often raise LDL cholesterol. Even foods rich in unsaturated fats, like walnuts or pecans, have minimal effect. Hormonal therapies, including thyroid hormone replacement and postmenopausal estrogen treatment, can moderately lower Lp(a), but these interventions are not recommended solely for cardiovascular risk reduction [7,8].

Despite these limitations, more intensive metabolic or dietary interventions may occasionally achieve substantial reductions in Lp(a). For instance, a 2024 case report described a 67-year-old vegan male who undertook a 10-day fast, consuming only water, followed by six weeks on a whole-food, plant-based diet. His Lp(a) dropped by 39%, from 236.3 nmol/L to 143.4 nmol/L - a reduction comparable to pharmacological interventions like PCSK9 inhibitors. This highlights that, in selected individuals, metabolic and dietary interventions may significantly reduce Lp(a), potentially through improved insulin sensitivity, reduced systemic inflammation, and modulation of hepatic lipoprotein synthesis. However, other fasting studies showed inconsistent results, indicating that these approaches may not be universally effective [9,10].

Pharmacologically, anti-PCSK9 therapies and lipoprotein apheresis are currently the most effective strategies for lowering Lp(a) and mitigating cardiovascular risk. Conventional lipid-lowering therapies, including statins, ezetimibe, and bempedoic acid, have minimal impact on Lp(a), which points to the importance of targeted treatment in high-risk individuals [9,10]. Emerging nucleic acid-based therapies, such as Zerlasiran, pelacarsen, olpasiran, and lepodisiran, show promise in significantly reducing Lp(a). Early trials with Zerlasiran demonstrated reductions exceeding 96% after single doses, with sustained reductions of 60%-90% following optimized dosing regimens. While these therapies are not yet approved, ongoing Phase 2-3 trials may provide effective targeted options for patients with markedly elevated Lp(a) [11].

Our case highlights the clinical importance of measuring Lp(a), particularly in individuals with a family history of premature cardiovascular disease. Current European guidelines recommend assessing Lp(a) at least once in adulthood to identify those at high inherited risk [12]. However, in routine clinical practice, Lp(a) testing remains uncommon, largely because there are few targeted treatment options. Family-based screening could facilitate earlier detection, especially as emerging therapies now offer the potential to meaningfully lower Lp(a). In our patient, despite optimal management of conventional risk factors, persistently elevated Lp(a) contributed to residual cardiovascular risk, prompting initiation of anti-PCSK9 therapy. This case underscores the need for individualized risk assessment and highlights how the availability of effective therapies may soon change clinical practice by encouraging earlier detection and intervention for patients with elevated Lp(a).

## Conclusions

Routine measurement of Lp(a) is essential for identifying inherited cardiovascular risk that may be overlooked by standard lipid assessment, particularly in individuals with premature disease or a strong family history. Early detection allows for more accurate risk stratification, individualized management, and closer clinical monitoring.

Promising results from emerging targeted therapies in clinical trials suggest that future treatment of elevated Lp(a) will become increasingly effective. As these therapies become available, greater clinician awareness and earlier screening are likely to improve risk reduction strategies and long-term cardiovascular outcomes.
